# Does mandible ramus height asymmetry affect postoperative skeletal stability in orthognathic surgery patients?

**DOI:** 10.1186/s40902-024-00442-9

**Published:** 2024-09-02

**Authors:** Jihun Cha, Kyuwon Park, Jaeyoung Ryu, Seunggon Jung, Hong-Ju Park, Hee-Kyun Oh, Min-Suk Kook

**Affiliations:** Department of Oral and Maxillofacial Surgery, School of Dentistry, Dental Science Research Institute, Chonnam National University, 42, Jebong-Ro, Dong-Gu, Gwangju, 61469 Republic of Korea

**Keywords:** Orthognathic surgery, Mandible, Facial asymmetry, Cone beam computed tomography

## Abstract

**Background:**

Relapses following orthognathic surgery have been reported to exceed 2% to 50%, depending on multiple factors. This study aimed to analyze the stability after orthognathic surgery in patients with mandibular ramus height asymmetry through 3D reconstruction using Cone-beam CT.

**Methods:**

This retrospective cohort study investigated patients who underwent mandibular setback surgery using bilateral sagittal split ramus osteotomy. Three-dimensional CT scans were taken at three different time points. Evaluation of the postoperative stability involved measuring changes in the x, y, and z axes as well as roll and yaw rotations of the mandible at specific landmarks (B point, mental foramen) on 3D CT scans obtained immediately after surgery and 6–12 months postoperatively. They were categorized into four groups based on bilateral mandibular height asymmetry through Asymmetry index (AI). The one-way ANOVA was implemented to compare the intergroup differences and Tukey's post hoc test was employed. Additionally, the Pearson correlation coefficient was also calculated.

**Results:**

A total of 24 patients were included in this study. The corresponding AI, representing the degree of asymmetry in both mandibles, were calculated as Group 1 was 1.25 ± 0.64%, Group 2 was 2.89 ± 0.47%, Group 3 was 5.03 ± 0.51%, and Group 4 was 9.40 ± 1.99%. The x-axis change in Group 4 was significantly larger at 1.71 mm compared to Group 1 at 0.64 mm. The mandibular roll, Group 4 showed a statistically significant increase at 1.33° compared to Group 1 at 0.35°. And there was a significant positive correlation observed between x-axis change and AI (*p* = 0.019), as well as between mandibular roll and AI (*p* = 0.025).

**Conclusion:**

After orthognathic surgery, stability was influenced by numerous factors, with the findings of this study suggesting that the degree of ramus height asymmetry in the mandible can be considered one contributing factor.

## Background

Orthognathic surgery is a surgical procedure aimed at correcting deformities and malocclusions, while significant advancements have occurred in osteotomy and surgical techniques. Sustaining stable outcomes post-surgery is pivotal, with skeletal stability a crucial consideration for long-term contentment [1].

As reported in the literature, relapse rates range considerably from 2.0% to 50.3%. Instances of relapse may lead to complications, such as malocclusion and compromised facial aesthetics [2]. The etiology of relapse is multifaceted, with factors impacting postoperative stability encompassing the extent of surgical movement, methods employed for the fixation of osteotomy segments, mandibular angles, adjustments to the proximal bone, soft tissues, and muscles, postoperative reconstruction of residual growth and mandibular condyles, patient age preoperatively, and the proficiency of the operating surgeon. Thus, extensive research has been conducted into factors predicting postoperative skeletal stability in orthognathic surgery [3–6].

Nevertheless, prevalent evaluations of skeletal stability predominantly rely on two-dimensional (2D) cephalometric radiographs, with limitations being raised. Challenges include inaccuracies in linear measurements, potential errors in skull rotation, and difficulties in comparing pre- and postoperative cephalometric radiographs due to restricted overlapping regions for superimposition. Significantly, three-dimensional (3D) analysis methods post-orthognathic surgery can evaluate the yaw, roll, and pitch, which are challenging aspects to discern through 2D cephalometric radiographs [7].

Mandibular asymmetry primarily contributes to facial asymmetry, manifesting as imbalances in the mandibular body or ramus height [8]. Congenital variations, such as the mandible under or overgrowing, may precipitate mandibular asymmetry. Discrepancies in volume or height between both mandibular bodies may induce interference between osteotomy segments during orthognathic surgery.

Despite extensive exploration of factors predicting postoperative skeletal stability in orthognathic surgery, there is a lack of research specifically addressing mandibular asymmetry exists. This study seeks to employ 3D cone-beam (CB) computed tomography (CT) reconstruction to compare patients with and without preoperative vertical asymmetry in mandible. This study aims to determine the effect on three-dimensional skeletal stability after mandibular orthognathic surgery by comparing patients with preoperative vertical asymmetry of the bilateral mandibular through reconstruction using 3D CBCT.

## Methods

This retrospective cohort study investigated patients who underwent mandibular setback surgery using bilateral sagittal split ramus osteotomy at the Department of Oral and Maxillofacial Surgery, Chonnam National University Hospital from January 2019 to December 2021. This study was approved by the institutional review board of Chonnam National University Hospital (CNUH-2023–349).

### Exclusion criteria

This study excluded patients with a history of previous orthognathic surgery, temporomandibular joint disorders (TMD), and those who had undergone previous trauma or cleft surgery. Patients were not excluded based on age, gender, or race.

### Variables

The predictor variable was mandible ramus height asymmetry (Asymmetry index, AI): Based on the AI, the 24 patients were classified into four groups: group 1 (AI < 2), group 2 (2 ≤ AI < 4), group 3 (4 ≤ AI < 6), and group 4 (AI ≥ 6).

### Surgery

All mandibular setback surgeries were performed by a single operator using bilateral sagittal split ramus osteotomy (BSSRO). After repositioning the mandible with a surgical splint, miniplates were used to fixate both sides. Intermaxillary fixation was applied with the surgical splint for 10 days postoperatively, after which patients were instructed to perform mouth opening exercises.

### Data collection

For each of the 24 patients had undergone at least six months of follow-up observations, and cone-beam computed tomography (CBCT) data were obtained before surgery (T0), immediately after surgery, and 6–12 months postoperatively using the DENTRI system (HDXWILL Inc., Seoul, Korea; 85 kVp; 8 mA).

**Fig. 1:**
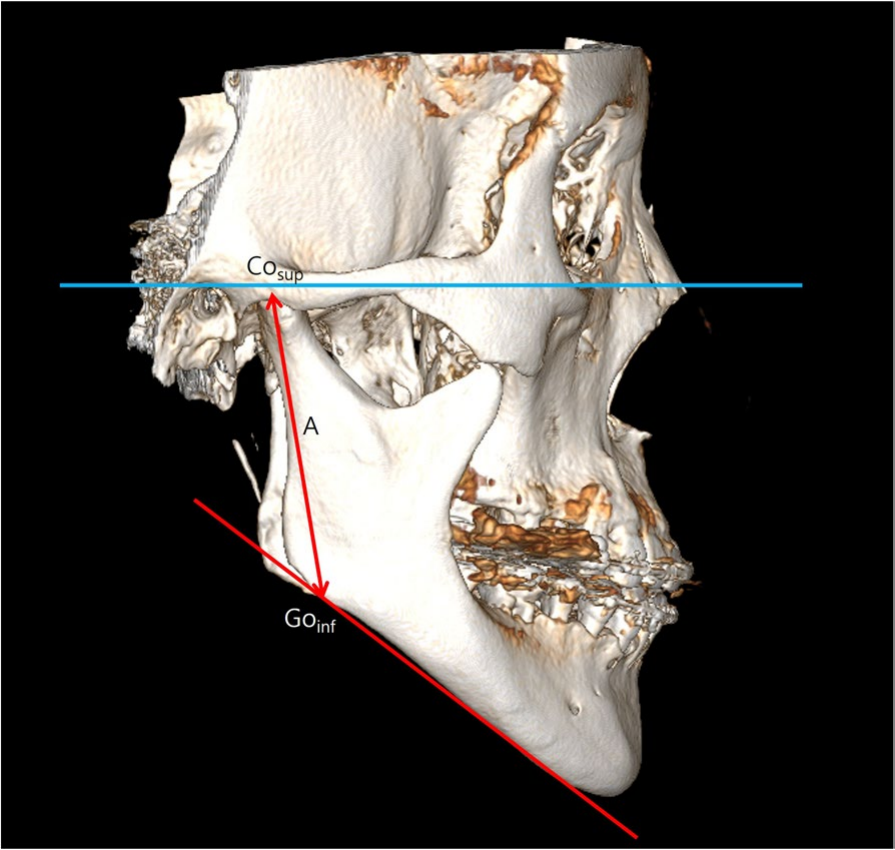
Definition of mandible ramus height through 3D-CBCT. After aligning with the FH plane, the distance is the straight line between the Cosup (the most superior point of the condyle head) and the Goinf (the tangent of the inferior border of the mandible meets the mandibular angle)

The preoperative CBCT scans (T0) were reconstructed into 3D models. The OnDemand3D program (CyberMed Inc., Seoul, Korea) was utilized to align the 3D coordinate system based on the Frankfort horizontal plane, using the orbitale and porion as reference points. The highest point of the mandibular condyle (Cosup) and the lowest point of the mandible angle tangent to the mandibular plane (Goinf) were then specified to define the vertical height of the mandible (Fig. 1).

To analyze mandibular asymmetry, the AI was employed using the formula AI = (|RHrt-RHlt|) / (RHrt + RHlt) × 100, where RHrt and RHlt represent the vertical height of the right and left mandibular sides, respectively [9].

**Fig. 2:**
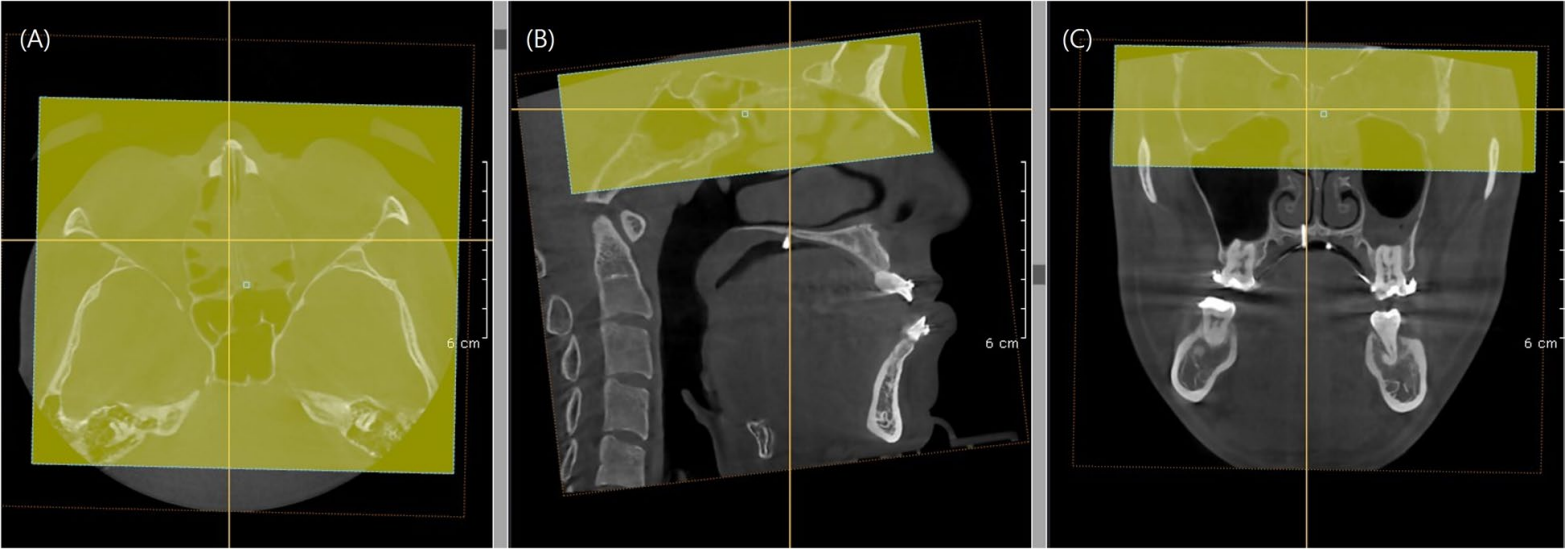
Superimposition of 3D-CBCT images with cranial base structures using the OnDemand3D software (CyberMed Inc., Seoul, Korea). The boxes indicate the areas of superimposition. (**A**): axial view; (**B**): sagittal view; (**C**): coronal view

To assess the skeletal stability after orthognathic surgery, postoperative images in Digital Imaging and Communications in Medicine (DICOM) format from two different time points immediately after surgery (T1) and 6–12 months after surgery (T2) were superimposed using the OnDemand3D program (CyberMed Inc., Seoul, Korea). The overlapping for the superimposition of T1 and T2 CT scans was performed by designating the cranial base, an area with no postoperative changes, as the region of interest in the axial, sagittal, and coronal views (Fig. 2). By utilizing the cranial base as a reference structure for superimposing 3D images, postoperative changes relative to this stable anatomical landmark can be accurately assessed [10].

**Fig. 3:**
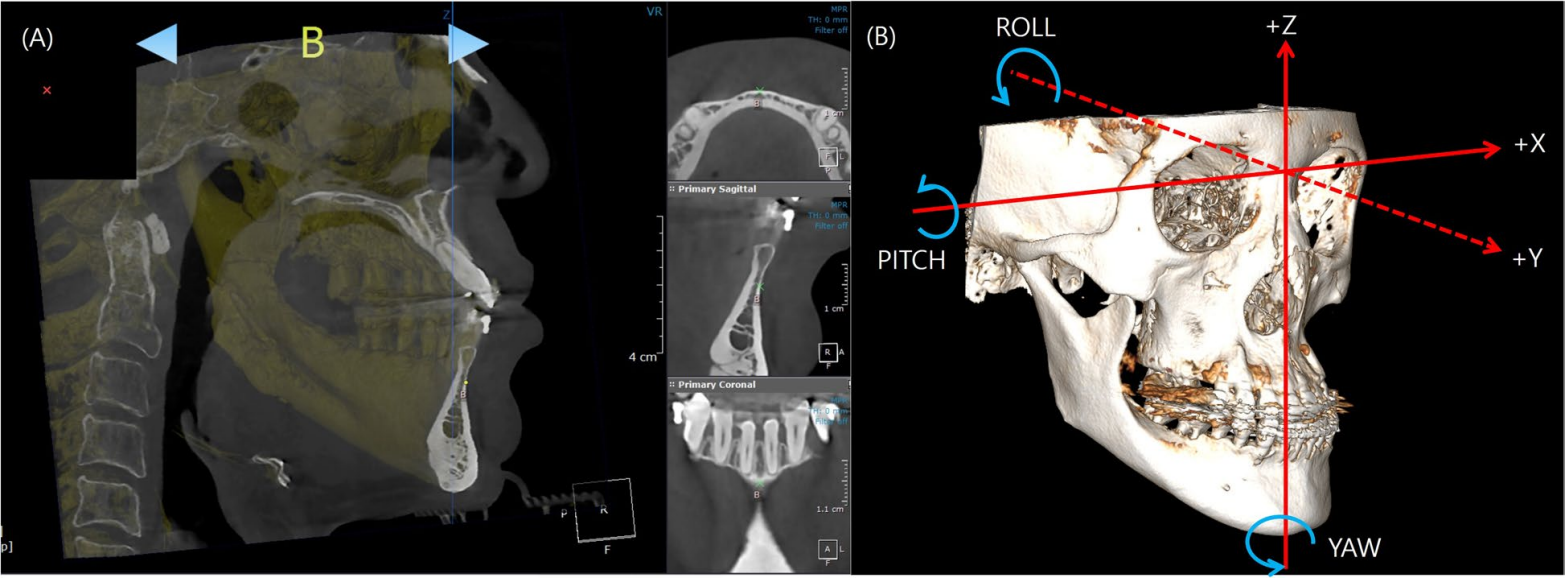
Alignment of the FH plane with the bilateral orbitale and pogonion points. The zero-point was set as Nasion. (**A**): Marking the B point on the 3D CT. (**B**): Definition of the x, y, and z axes, as well as yaw, pitch, and roll on the 3D CT. X-axis: ( +) left, (-) right; Y-axis: ( +) anterior, (-) posterior; Z-axis: ( +) superior, (-) inferior

After superimposition to the T1 and T2 CT data, the alignment was performed based on the Frankfort horizontal plane, with the zero-point set at Nasion. Subsequently, the coordinate systems for the x, y, and z axes were established (Fig. 3).

### Data analysis

The following measurement points were defined to assess skeletal stability after orthognathic surgery using the 3D-CT images, and the changes in yaw and roll rotation of the mandible were determined (Table 1) [11].

**Table 1:** Description of the landmarks and measurements on the 3D-CT

Landmark	Definition
Nasion	The most anterior point of the frontonasal suture
Orbitale	The most inferior point of the infraorbital margin of the orbit
Porion	The most superior point of the external auditory meatus
B point	The midpoint of the greatest concavity of the anterior border of the symphysis
Rt mental foramen (MFrt)	Center of the right mental foramen of the mandible
Lt mental foramen (MFlt)	Center of the left mental foramen of the mandible
**Angles**	**Measurement**
Mandibular yaw	The angle between the mental line (MFrt-MFlt) and coronal plane
Mandibular roll	The angle between the mental line (MFrt-MFlt) and sagittal plane

**Fig. 4:**
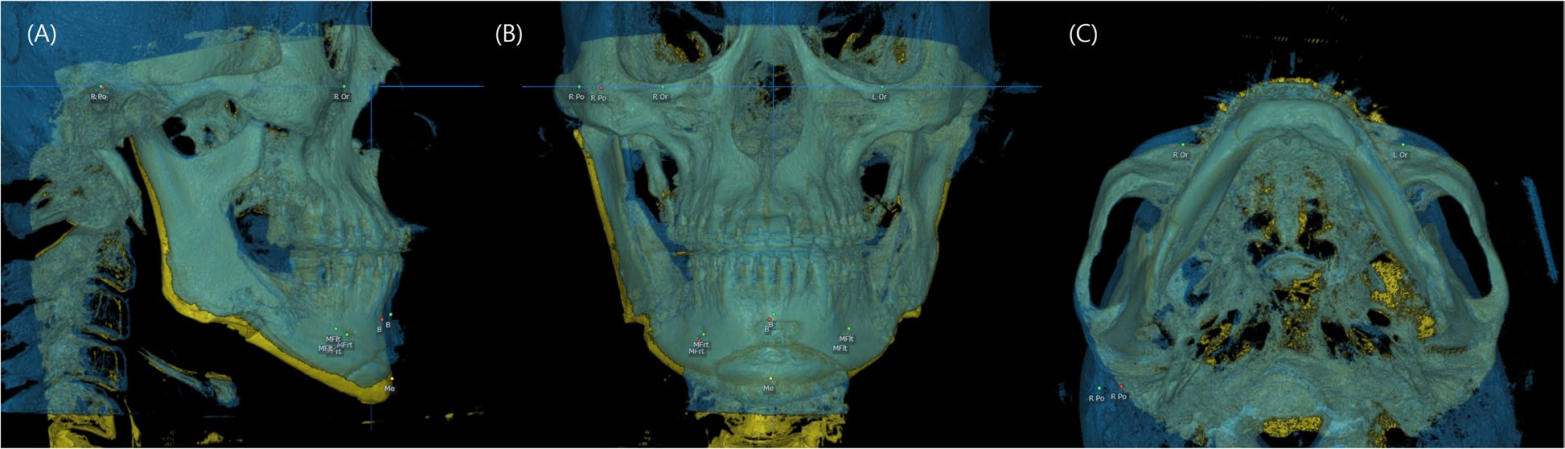
A superimposed 3D-CBCT directly after surgery (yellow) and 6–12 months after surgery (blue). The position change of the B point and mental foramen. (**A**): lateral view right; (**B**): frontal view; (**c**): submentovertex view

**Fig. 5:**
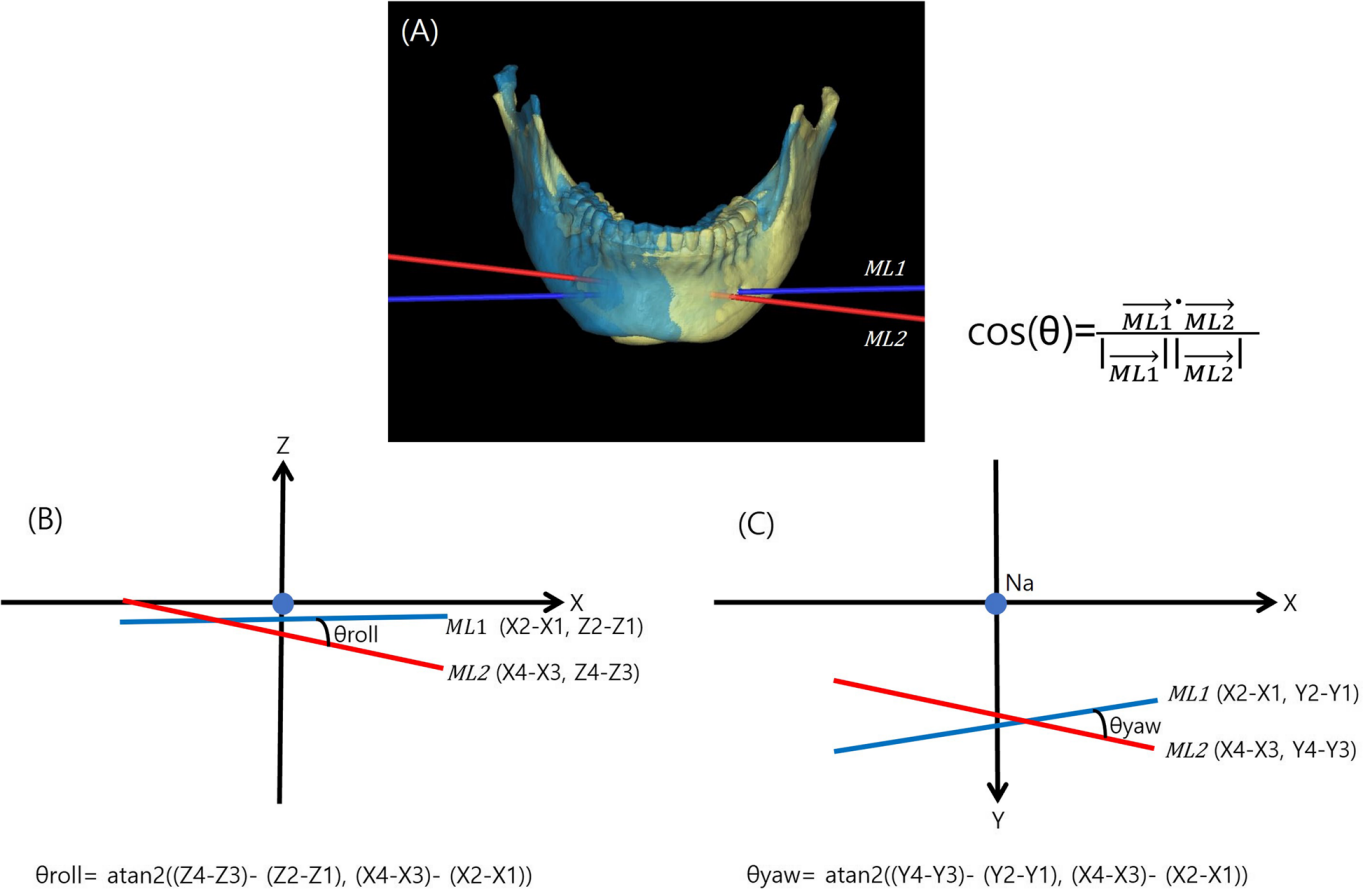
(**A**): Three-dimensional representation of the mental foramen line according to postoperative changes between T1 and T2, Yellow: T1; Blue: T2. (**B**): Calculation of roll movement using ML1 and ML2, (**C**): Calculation of yaw movement using ML1 and ML2. ML1: Mental foramen line 1 in T1; ML 2: Mental foramen line 2 in T2

Using the overlaid 3D images, the positional changes of the defined measurement points were measured in a 3D coordinate system (Fig. 4). By utilizing the positional changes in the altered measurement points, data on the changes in the x-axis, y-axis, and z-axis directions of the mandible were obtained immediately after surgery (T1) and 6–12 months after surgery (T2). The data were compared to ascertain if a significant relapse occurred in each direction. To confirm the yaw and roll after surgery, the rotation angles around the z-axis and y-axis of the mental foramen line were calculated before surgery and after surgery using the atan2 ((y4-y3)-(y2-y1), (x4-x3)-(x2-x1)1) and atan2 ((z4-z3)-(z2-z1), (x4-x3)-(x2-x1)) functions, respectively (Fig. 5) [12].

To assess intra-observer variation, one observer performed repeated superimposition, landmark marking, and coordinate measurements in 24 patients at 2-week intervals. This involved six landmarks, three coordinates per landmark, total 432 measurements across the patients. The intraclass correlation coefficients from the two-way random effects model were calculated to be 0.915 with statistical significance.

### Statistical analysis

Each group satisfied the assumption of homogeneity of variances, while normality was confirmed using the Shapiro–Wilk test (*p* > 0.05). For the statistical analysis, a one-way ANOVA was conducted using the R 4.3.1 statistical software (R Development Core Team, Vienna, Austria) to identify any significant differences among the four AI-classified groups in the relapse amounts in the x-, y-, and z-axis directions of B point and the yaw and roll rotation amounts following surgery. Following the analysis, Tukey's post hoc test was employed to identify the specific groups with significant differences.

Additionally, the Pearson correlation coefficient was calculated to determine if a significant correlation existed between the vertical asymmetry of the mandible and the degree of relapse after surgery.

## Results

A total of 24 patients were included in this study, and demographic data for the four groups defined based on the AI are summarized (Table 2). The follow-up period after surgery for all patients was at least 6 months.

**Table 2:** Demographic data of the patients in each group

	Group 1 (*n* = 9)	Group 2 (*n* = 5)	Group 3 (*n* = 4)	Group 4 (*n* = 6)	*p*-value
Sex					
Male (*n* = 12)	6	4	1	1	
Female (*n* = 11)	3	1	3	5	0.09^a^
Age					
Mean ± SD (years)	20.78 ± 1.93	28.2 ± 6.94	21.5 ± 6.10	23.0 ± 6.66	0.18^b^
Ramus difference (│Rt—Lt│)					
Mean ± SD (mm)	1.72 ± 0.89	3.95 ± 0.58	6.55 ± 0.77	9.40 ± 2.34	
Asymmetry index (AI)					
Mean ± SD (%)	1.25 ± 0.64	2.89 ± 0.47	5.03 ± 0.51	9.40 ± 1.99	

^*a*^*Chi-square test*

^*b*^*One-way ANOVA test*

The vertical height difference in mandible for the four groups was measured as follows: Group 1: 1.72 ± 0.89 mm, Group 2: 3.95 ± 0.58 mm, Group 3: 6.55 ± 0.77 mm, and Group 4: 9.40 ± 2.34 mm. The corresponding AI, representing the degree of asymmetry in both mandibles, were calculated as 1.25 ± 0.64%, 2.89 ± 0.47%, 5.03 ± 0.51%, and 9.40 ± 1.99%, respectively.

**Fig. 6:**
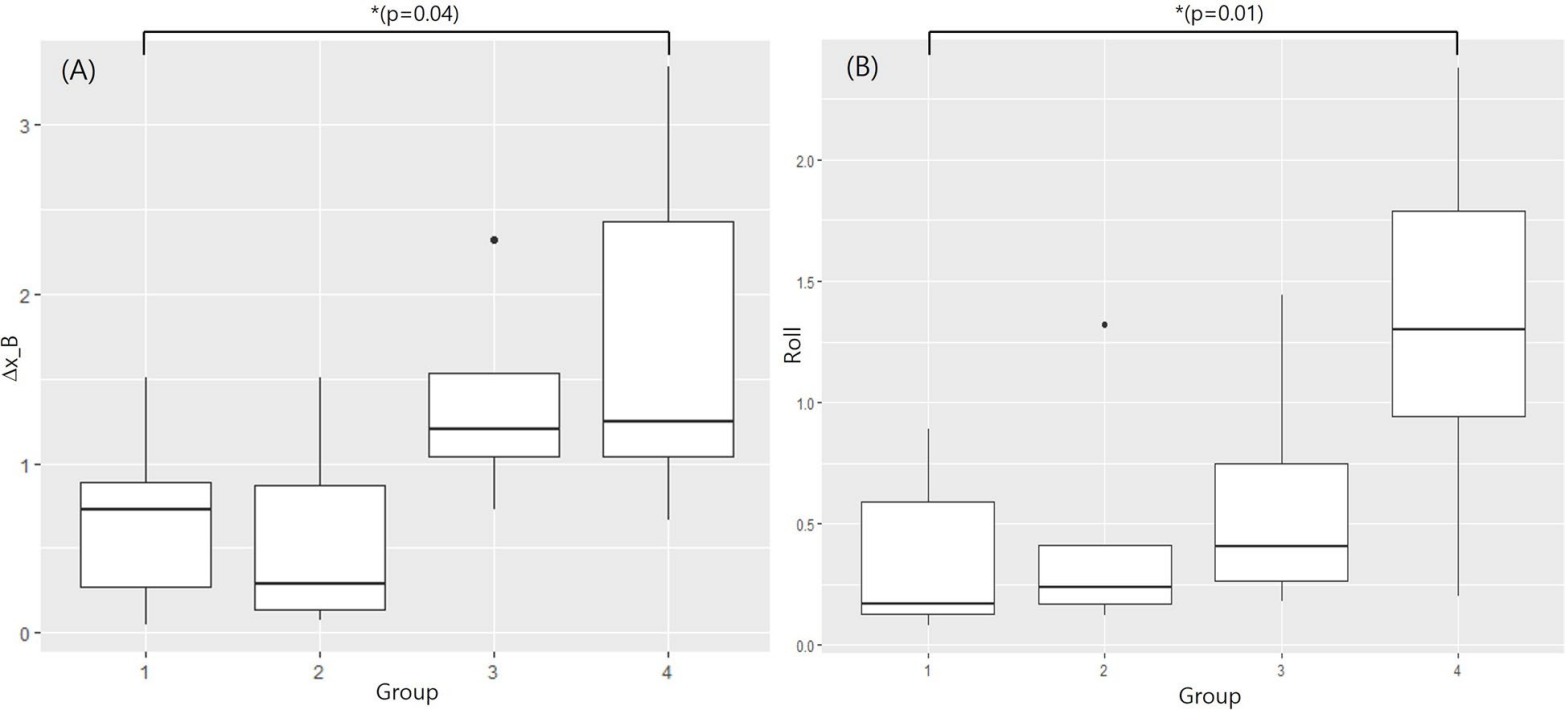
Box plot showing the postsurgical changes (T1–T2) in the superimposition of CT in the 4 groups. (**A**): ∆x movement of B point; (**B**): the mandibular roll movement

Table 3 presents the changes in B point after orthognathic surgery in the x-axis (lateral), y-axis (anteroposterior), z-axis (vertical) directions, and total 3D displacement (∆B point) as well as the yaw and roll rotation changes in the mandible immediately after surgery (T1) and 6–12 months after surgery (T2) for Groups 1, 2, 3, and 4. Using Tukey's multiple comparison test after one-way ANOVA, statistically significant differences were observed in the x-axis (lateral) change and mandibular roll between Group 1 and Group 4 (*p* = 0.031 and *p* = 0.016, respectively). The x-axis change in Group 4 was significantly larger at 1.71 mm (SD, 1.09 mm) compared to Group 1 at 0.64 mm (SD, 0.46 mm) (*p* < 0.05). Additionally, in the mandibular roll, Group 4 showed a statistically significant increase at 1.33° (SD, 0.77°) compared to Group 1 at 0.35° (SD, 0.31°) (*p* < 0.05) (Fig. 6).

**Table 3:** Postoperative stability result of the 3D assessment between postoperative changes (T1 and T2) in the mandible

	Group 1		Group 2		Group 3		Group 4		*P*-value	Post hoc
	Mean	SD	Mean	SD	Mean	SD	Mean	SD		
Translational movements (mm) of B point										
Lateral (∆x)	0.64	0.46	0.58	0.61	1.37	0.68	1.71	1. 09	0.031^*^	4 > 1^*^
Anteroposterior (∆y)	0.21	1.30	0.71	1.06	2.25	0.85	1.05	2.36	> 0.05	-
Vertical (∆z)	1.22	1.05	2.18	0.93	0.36	1.58	0.48	0.71	0.048^*^	> 0.05
Variable (∆B point)	2.19	1.01	2.18	0.36	3.38	1.39	2.84	0.90	> 0.05	-
Rotational movements (°) of Mental line (MFrt-MFlt)										
Mandibular yaw	0.83	0.54	0.49	0.39	1.79	0.84	1.54	1.33	> 0.05	-
Mandibular roll	0.35	0.31	0.45	0.50	0.61	0.57	1.33	0.77	0.016^*^	4 > 1^*^

*One-way ANOVA and Tukey's *post hoc* tests.* ∆x: absolute value; ∆y: anterior ( +), posterior (-); ∆z: superior ( +), inferior (-); yaw: absolute value; roll: absolute value

^*^p < 0.05

Table 4 presents the correlation analysis results between the AI and the changes in the x-axis, y-axis, and z-axis of the B point and the yaw and roll rotation changes in the mandible. A higher AI value is significantly positively correlated with a greater postoperative change in the x-axis of the B point (r = 0.47, *p* < 0.05). Additionally, a higher AI value is significantly positively correlated with a greater postoperative roll rotation movement in the mandible (r = 0.46, *p* < 0.05).

**Table 4:** Correlation between AI and variables

	B point						yaw (∘)		roll (∘)	
	∆x (mm)		∆y (mm)		∆z (mm)					
	r	*P*-value	r	*P*-value	r	*P*-value	r	*P*-value	r	*P*-value
Asymmetry index (AI)	0.47	0.019^*^	-	0.099	-	0.643	0.39	0.059	0.46	0.025^*^

Pearson correlation coefficient, *p < 0.05

**Fig. 7:**
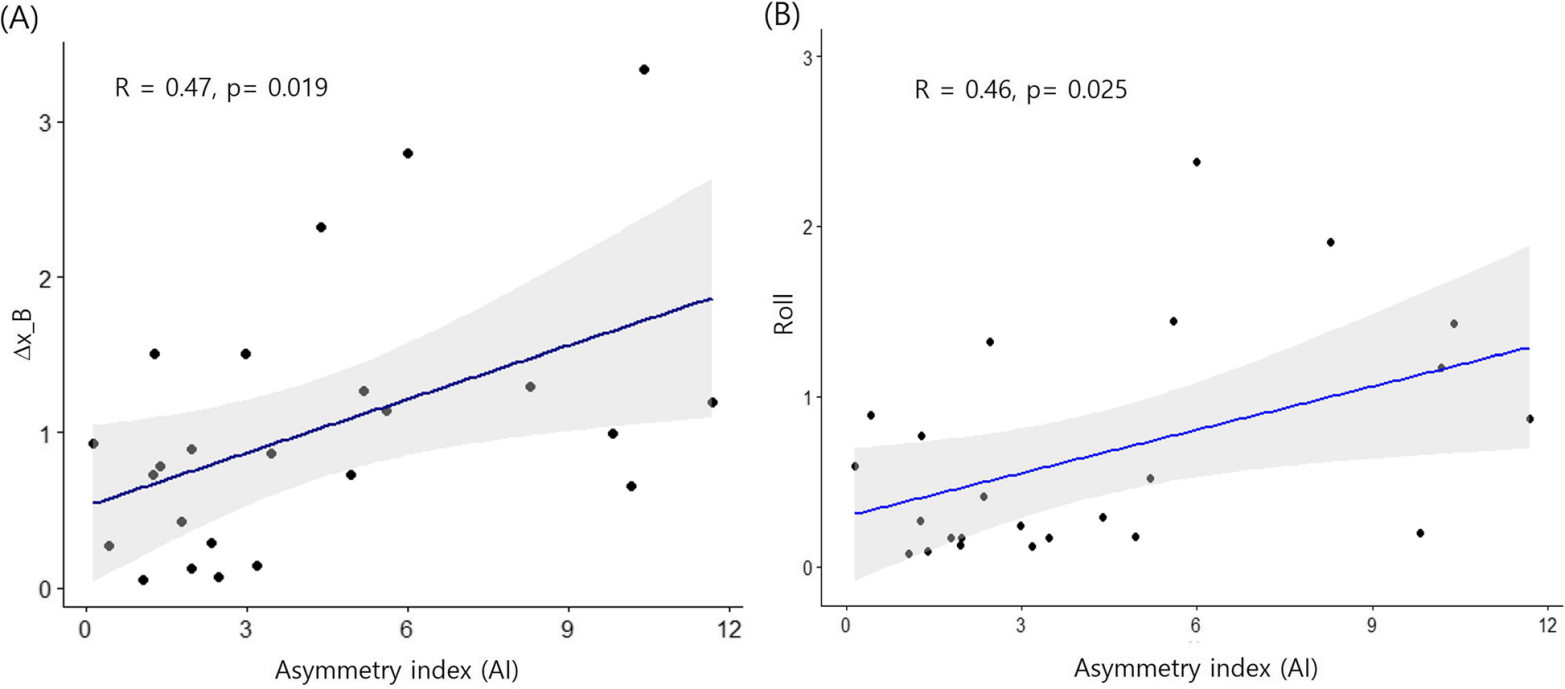
A scatter plot showing the relationship between the AI and the extent of postsurgical mandibular changes (T1–T2). (**A**): ∆x of B point; (**B**): mandibular roll movement

The correlation between the postoperative x-axis change and the roll rotation in the mandible with AI is visually represented in Fig. 7.

## Discussion

Mandibular setback surgery can be performed as an independent procedure to achieve posterior movement by the mandible or as part of a combined orthognathic surgery involving maxillary surgery. Among the complications, skeletal stability after orthognathic surgery is crucial for achieving satisfactory long-term results. Various studies are actively investigating factors for predicting relapse, including maxillary surgery, the need for presurgical orthodontic treatment, direction and amount of mandibular movement, surgical technique, and the duration of intermaxillary fixation. However, conflicting results are still being reported in the literature [13–15].

Kim et al. attributed early relapse (within 6 months post-surgery) to mandibular condyle position changes and bone cut slippage [16]. In contrast, Tong Xi et al. linked late relapse (6–12 months post-surgery) to factors such as surgical extent, technique, and mandibular condyle resorption [17].

Valls-Ontañón et al. described skeletal stability after orthognathic surgery as a state without relapse, indicating no undesired movements in the sagittal, transverse, and vertical directions at the measurement points on the 3D radiographic images [18].

Conversely, Habets et al. defined vertical asymmetry of the mandible in panoramic radiographs as a significant difference between the right and left sides exceeding 6% [9]. McCrea et al. defined an AI exceeding 3% as indicative of a structural difference of 6% or more in mandibular length, establishing 3% as the threshold for detecting asymmetry in mandibular length [19].

Many studies on post-orthognathic surgery relapse have relied on 2D lateral cephalometric radiographs for evaluation. However, these measurements can be prone to errors due to variations in X-ray beam angles, challenges in replicating head position, and inaccuracies in distance measurements. In contrast, 3D analysis enables assessment of skeletal changes across the x-axis, y-axis, and z-axis with minimal distance measurement errors [20, 21]. While many post-orthognathic surgical relapse studies have primarily focused on the sagittal plane, it is widely acknowledged that relapse can also occur in the vertical and transverse planes [22].

For CT overlap in 3D analysis, Rania et al. suggested that voxel-based image registration is an accurate and reproducible semiautomatic 3D-CBCT overlap method commonly used for overlapping regions such as the anterior cranial base or zygomatic arch [23]. This study conducted a 3D analysis using CBCT scans overlapping the anterior cranial base region. Additionally, to minimize the impact during surgery, bilateral mental foramina, a region minimally influenced during surgery, were measured as reference points to analyze changes in B point and rotational movements.

The purpose of this study was to evaluate the stability after mandibular orthognathic surgery in patients with significant vertical asymmetry of the mandible. Three-dimensional CT superimpositions were used to assess the postoperative stability of mandibular movement in three dimensions, as well as yaw and roll movements.

The results indicated that, in the x-axis direction of lateral movement, an AI of > 6% in Group 4 exhibited significantly greater lateral movement than Group 1 during the 6–12 months post-surgery period. No significant differences were observed between the four groups in the y-axis, z-axis, and 3D movement of the B point. When comparing mandibular roll direction rotational movement, Group 4 showed a significantly larger mandibular roll movement compared to Group 1 during the 6–12 months post-surgery period. This suggests that in cases where the AI indicates a bilateral mandibular asymmetry of 6% or higher, significant lateral and mandibular roll movements may occur post-surgery, signifying potential decreased skeletal stability in those directions.

The correlation analysis between the vertical asymmetry index of bilateral mandibles and the movement of the B point in the x, y, and z axes, as well as the yaw and roll rotational movements of the mandible, revealed a significant positive correlation between the AI and the increase in the x-axis movement of the B point. Additionally, a significant positive correlation was observed between the AI and the magnitude of the mandibular roll rotational movement. This indicates that as the asymmetry in the bilateral mandibles becomes more pronounced, there is a decrease in postoperative skeletal stability, particularly in lateral movement and mandibular roll rotational stability.

The potential cause of these results is the interference difference in the centrically based bone segment during surgery due to the asymmetry in the mandibles. During orthognathic surgery, the inevitable interference between the centrically based bone segment and the postoperative position of the mandible's lateral movement occurs. To minimize this interference and enhance postoperative skeletal stability, the surgical approach involves removing the interfering bone segment to allow for passive contact during surgery. However, the asymmetry in the mandibles may result in different interference amounts between the left and right sides.

In other literature, various factors contributing to the relapse of menton deviation in patients with facial asymmetry have been reported in different studies. Commonly recognized factors include differences in muscular activity due to asymmetrical amounts of set-back on the left and right sides, as well as condylar deviation [24, 25]. To prevent these factors, minimizing interference between the proximal and distal segments to prevent condylar deviation is crucial [26]. Additionally, it is believed that counterclockwise rotation of the mandible's proximal segment, as well as reducing the lengths of the masseter and pterygoid muscles, can prevent relapse, as previously known. Moreover, it is thought that securely detaching the pterygomasseteric muscle from the bone or lingual short cut technique could also prevent relapse.

In this study, patients with severe asymmetry, such as those in group 4, also exhibited canting of the maxilla. However, 1-jaw surgery was performed based on patient preference, resulting in satisfactory outcomes postoperatively. Nevertheless, it is hypothesized that if 2-jaw surgery had been performed to correct the canting in severe asymmetry cases, it might have yielded different results in terms of postoperative stability.

This study focused solely on assessing postoperative changes within a one-year. Therefore, further research is necessary to investigate long-term stability and potential impacts of factors like mandibular setback extent and inclusion of bimaxillary surgery on postoperative outcomes. Another limitation is the small sample size of 24 cases, which constrained the study's ability to divide groups effectively. Additionally, the lack of prior research on postoperative instability based on the asymmetry index limited the study, as the index was arbitrarily classified into four groups. Future studies with larger sample sizes are required to address these limitations comprehensively.

## Conclusion

While many factors contribute to postoperative stability following orthognathic surgery, the results of this study suggest that the degree of vertical asymmetry in the mandibles can be considered an influencing factor. In further studies, evaluation of skeletal stability after orthognathic surgery in longer follow-up period is necessary with prospective study.

## Data Availability

Please contact the author for data requests.

## Abbreviations

BSSRO: Bilateral sagittal split ramus osteotomy
CBCT: Cone-beam CT
AI: Asymmetry index
FH: Frankfort horizontal plane
2D: Two-dimensional
3D: Three-dimensional
DICOM: Digital Imaging and Communications in Medicine
